# Effects of COVID-19 Pandemic on Metabolic Status and Psychological Correlates of a Cohort of Italian NAFLD Outpatients

**DOI:** 10.3390/nu15061445

**Published:** 2023-03-16

**Authors:** Silvia Ferri, Bernardo Stefanini, Marta Minguzzi, Simona Leoni, Roberta Capelli, Alice Secomandi, Rusi Chen, Chiara Abbati, Ernestina Santangeli, Katia Mattarozzi, Piscaglia Fabio

**Affiliations:** 1 Division of Internal Medicine, Hepatobiliary and Immunoallergic Diseases, IRCCS Azienda Ospedaliero—Universitaria di Bologna, 40138 Bologna, Italy; 2 Department of Medical and Surgical Sciences, University of Bologna, 40126 Bologna, Italy

**Keywords:** NAFLD, COVID-19, metabolic syndrome, psychological distress, behavioral approach

## Abstract

**Simple Summary:**

One out of four patients with NAFLD and metabolic syndrome is also affected by psychological distress. In this study, we evaluated the impact of a stressful contingency such as the COVID-19 lockdown on a cohort of Italian patients affected by NAFLD. Our data confirmed the association between psychological distress and NAFLD. They also showed how psychological suffering was associated with a worse efficacy of the behavioral therapeutic approach in patients with NAFLD.

**Abstract:**

Non-alcoholic fatty liver disease (NAFLD) is a potentially progressive condition characterized by the presence of fat in more than 5% of hepatocytes, representing the hepatic expression of metabolic syndrome (MetS). A reduction of at least 5–7% in initial body weight improves the metabolic profile underlying NAFLD. The aim of our study was to evaluate the effects of the COVID-19 lockdown on a cohort of non-advanced NAFLD Italian outpatients. We identified 43 patients with 3 available time point visits in our center: first visit (T0) when behavioral indications aimed at controlling MetS were provided, a pre-COVID visit (T1) and a post-COVID visit (T2). During the lockdown, an online compilation of validated psychological tests (SRQ-20, EQ5D, SF-12 and STAI) and a specifically formulated questionnaire for NAFLD was presented to our cohort and completed by 14 consenting patients. Patients who had lost more than 5% of the initial weight at T1 (9 subjects, 21%) maintained the results even at T2, with an overall reduction in BMI and liver stiffness; patients who had not lost the desired weight at T1 (34 subjects, 79%) displayed a further increase in BMI and visceral adiposity at T2. Of interest is that patients in the latter group reported signs of psychological suffering. Our data demonstrated that the setting of good counseling was effective in controlling the metabolic disorder underlying NAFLD in our cohort of outpatients. Given the need for patients to play an active role in the behavioral therapy for NAFLD, we advocate that a multidisciplinary approach be adopted, including a psychological support to obtain the best results over time.

## 1. Introduction

Non-alcoholic fatty liver disease (NAFLD) is characterized by excessive hepatic fat accumulation, defined as the presence of steatosis in >5% of hepatocytes, detectable by imaging techniques or histology, and includes a spectrum of disorders ranging from simple fatty liver (NAFL) to non-alcoholic steatohepatitis (NASH). This condition includes the risk of fibrosis, cirrhosis and hepatocellular carcinoma (HCC). Obesity, insulin-resistance or type 2 diabetes mellitus, arterial hypertension and dyslipidemia, all key elements of the so-called metabolic syndrome (MetS), are the most relevant conditions related to NAFLD [1]. The treatment of NAFLD and NASH is primarily aimed at reducing the progression of liver disease to cirrhosis and HCC and, consequently, NASH-related mortality. In the absence of an approved beneficial pharmacological therapy, the use of an integrated lifestyle approach to correct the multiple imbalances that underpin the MetS as a whole, including chronic inflammation, represents the therapeutic cornerstone for NAFLD/NASH patients. Weight loss is the first and most effective strategy for the management of MetS-associated NAFLD [2]. A weight loss >5% (in obese patients 7–10%) compared to baseline improves hepatic steatosis, inflammation and fibrosis in NAFLD patients [3,4]. The most recommended dietary regimen for NAFLD is based on the Mediterranean diet [5,6], characterized by a behavioral approach to nutrition based on the seasonality and sustainability of products and on a culture of preparing food and consuming the meal in a convivial manner together with regular physical activity and adequate rest [7]. In particular, Mediterranean diet is based on a great reduction in the intake of highly processed food (such as sweets, sugars, sugary drinks, processed meat, ready and pre-cooked foods) in favour of complex carbohydrates (>40% of daily calories, mainly from whole grains, legumes and fibres), fats (35–45% of the total energy, mainly from mono and poly-unsaturated fatty acids) and proteins from vegetal and animal sources, with a preference for dairy products, eggs, fish and white meat. Adequate intakes of vitamins E, C and phenolic compounds, contained in nuts, seeds, vegetable oils, fruits, vegetables, coffee and tea, can protect the liver from NAFLD-related damage and the consumption of up to one glass of wine per day, as a part of a Mediterranean diet, may be considered in patients with non-advanced NAFLD [8,9,10]. Physical inactivity, which is common in patients with NAFLD, represents an independent risk factor for disease development and progression [11]. On the contrary, favouring glucose entry into myocytes, even in cases of insulin-resistance condition [12], physical activity is effective in reducing liver fat together with metabolic comorbidities, even without a significant weight loss [13]. Aerobic activity and resistance exercise display different helpful characteristics and the greatest benefit comes from the combination of the two, so that guidelines recommend at least 150 min of moderately intense exercise per week, divided into 3–5 sessions, combined both aerobic activity and resistance exercises [14]. 

Given the need for patients to play an active role in the behavioral therapy for NAFLD, it is important to assess also their psychological well-being, which appears to be relevant to adopting the required lifestyle changes. Up to 25% of patients with NAFLD/NASH have depression and/or eating disorders, with a connection between the two conditions: on one hand MetS can be associated with fatigue and exhaustion, leading to withdrawal from social life, feelings of guilt and inability to manage activities of daily living. On the other hand, during psychological distress, eating can become a means of compensation for negative feelings and frustration, leading to weight gain [15]. As a result, higher NAFLD/NASH frequency and severity have been associated with major depression and generalized anxiety disorders, bipolar disorder and schizophrenic disease [16,17], but also with mild psychological uneasiness characterized by low awareness and high neuroticism but that does not reach the level of overt psychological disorders [18].

The molecular bases for this close association are still under investigation, but some proposed mechanisms include oxidative stress and mitochondrial dysfunction, leading to ATP depletion, both in the liver and in the brain, which causes liver damage and neuroinflammation; of interest is that some of the agents stimulating the antioxidant defenses of the mitochondria, already proposed for use as support therapy in NAFLD (such as alpha-lipoic acid, vitamin D, vitamin E, coenzyme Q10 and fibrates), are under investigation in various psychiatric disorders [19,20]. In addition, a Western diet with excessive caloric intake mainly due to ultra-processed food, fructose, saturated fats and highly refined carbohydrates, a diet strictly related to NAFLD pathogenesis, has been associated with mental health disorders through their effects on inflammation, oxidative stress, epigenetic modifications and neuroplasticity [21]. 

The wide and uncontrolled spread of SARS-CoV-2 in Italy in winter 2020 forced the authorities to declare an emergency status, characterized by a complete lockdown and a massive temporary conversion of medical resources for use in COVID-positive patient care [22]. Consequently, a sudden and dramatic limitation to sociality and physical activity and a reduced possibility to regularly follow outpatients in hospital occurred. These measures would be expected to impact on NAFLD patients via a combination of changes in physical activity, diet quality, and psychological wellbeing. A reduction in physical activity and quality of diet was reported during the lockdown, with a return to normal habits after the end of the period of restrictions. Despite the absence of relevant changes in body composition, a significant increase in glucose, total and LDL cholesterol blood levels were found in healthy adults [23]. As for NAFLD patients, a study performed in 41 Italian overweight and obese outpatients [24], evaluated before and immediately after the end of the lockdown period, documented a weight gain in 70% of patients, with a reversed trend compared to previous evaluations. Weight gain was more evident in those subjects who did not exercise and did not adhere to the Mediterranean diet during the lockdown period. 

The aim of this study was to describe the metabolic conditions and psychological distress in NAFLD patients during the burden of the COVID-19 pandemic lockdown and to evaluate their association with the capacity to achieve and maintain effective healthy lifestyle changes. 

## 2. Materials and Methods

We decided to analyze patients in the charge of our liver outpatient clinic with defined NAFLD according to EASL guidelines [1] (fatty liver at ultrasound, HSI—hepatic steatosis index >30 and exclusion of other well-known causes of fatty liver, namely significative alcohol consumption corresponding to more than 2 units per day for females and 3 units per day for males, steatogenic drugs and liver storage diseases; patients with current or previous history of HBV or HCV infections were also excluded). In order to better focus on the impact of the emergency lockdowns in facing the COVID-19 pandemic, we decided to limit our study to 43 patients meeting the following inclusion criteria, having had: a first visit in 2017–2018 (T0), a subsequent pre-pandemic visit in 2019 (T1), a programmed visit between March and June 2020, missed due to the pandemic restrictions, and a post-pandemic visit after September 2020 (T2). Patients with severe disease (decompensated cirrhosis and/or hepatocellular carcinoma) were excluded from this study as they were also seen in the outpatient clinic during the lockdown period. In keeping with the aim of the present study, which is to investigate the capacity to maintain lifestyle changes despite the restrictions of lockdown period, we subgrouped patients according to whether they had been able to modify their lifestyle to reach the goal of more than 5% weight loss before the occurrence of the COVID-19 pandemic. 

At T0, all patients underwent an accurate physical examination, recording height, weight and waist measurements without shoes and with light clothing. Arterial blood pressure was recorded after a five-minute resting period. A full physiological and pathological history was recorded. A diagnosis of type 2 diabetes mellitus was declared if the disease had been previously diagnosed or, in case of history of ongoing or previous pharmacological treatment for diabetes or fasting glucose >116 mg/dL, was found in at least two consecutive determinations. Insulin resistance was defined as when a patient had HOMA (homeostasis model assessment) index score >2.5 in at least two determinations. Dyslipidemia was defined for LDL >100 mg/dL and/or triglycerides >150 mg/dL and/or HDL <40 mg/dL for men and <50 mg/dL for women and/or for use of at least one lipid-lowering drug. Arterial hypertension was defined as previously diagnosed, ongoing or previous pharmacological treatment for hypertension or when a blood pressure >135/90 mmHg was found on two separate occasions. Weekly physical activity during the month before the visit was evaluated using a quantitative questionnaire: no physical activity at all was classified as 0, no leisure-time physical activity but active life as doing housework was classified as 1 (scarce), up to 90 min of leisure-time physical activity of at least moderate intensity was classified as 2 (moderate) and more than 90 min was classified as 3 (good). Patients were also divided into non-smokers and smokers according to whether they had never or had ever smoked. For the latter group, exposure to cigarette smoke was quantified in pack years (one pack-year corresponds to an amount of twenty cigarettes smoked every day for one year).

At T0, all patients were given dietary advice following the Mediterranean approach and encouraged to increase their physical activity up to 150 min/week of aerobic and/or anaerobic activity of moderate intensity (Table S1 in Supplementary Materials). Medications such as metformin, lipid and blood pressure-lowering therapies were prescribed when needed.

At the three time points, a complete anamnestic, physical, laboratory and ultrasound evaluation was performed. Anthropometric parameters were evaluated in all patients: weight, height, BMI (weight in kg/height in meters^2^, with the following ranges: normal weight = from 18.5 to 24.9; overweight = from 25 to 30, first degree obesity = from 30.1 to 34.9, second degree obesity = from 35 to 39.9, third degree obesity ≥ 40) and waist circumference.

At each visit, blood tests executed in the previous 30 days were evaluated: white blood cells, hemoglobin, MCV, platelets, proteins, albumin, bilirubin, GOT, GPT, GGT, alkaline phosphatase, glycemia, insulin, glycosylated Hb, total, HDL and LDL cholesterol, triglycerides, urea, TSH reflex end vitamin D were studied. An abdominal ultrasound was also performed, considering: echogenicity of the liver, thickness of cutaneous and visceral adipose tissue, “B-mode hepato-renal sonographic index” (H/R s-index) [25] and liver stiffness measurement through “2-dimension Shear Wave Elastography” (2D-SWE) using the device Supersonic Aixplorer [26]. Finally, the following scores were calculated: NFS, FIB-4 and HOMA index.

At T1 and T2, all the anamnestic, laboratory and ultrasound characteristics examined during the first visit were re-evaluated. In addition, patients were asked about adherence to diet and physical activity.

In order to investigate psychological distress during the lockdown period, all patients were contacted by phone by one physician of our clinic. Participants were preliminarily requested to give their informed consent to complete an online interview programmed with Qualtrics software. Consenting patients were asked to fill a series of validated questionnaires which were aimed at assessing psychological wellbeing and quality of life. Patients could only be contacted by phone at the time of the study as during lockdown period medical facilities were accessible only in case of emergency or severe life-threatening diseases. 

More specifically, the following validated questionnaires were used:

The Self-Reporting Questionnaire-20 (*SRQ-20*) is a 20-item questionnaire that includes binary (yes = 1; no = 0) questions about physical and psycho-emotional symptoms that may have occurred in the past 30 days. A positive screening for mild psychiatric disorders was considered to be given if the questionnaire had a score of 7 or higher, which was taken as a sign of current mental distress [27].

The State—Trait Anxiety Inventory (*STAI-Y2*) was used to index trait anxiety as a stable and persistent emotional state able to affect the habitual way of responding to external stimuli. The *STAI-Y2* is composed of 20 questions, where the participant assesses how different statements fit his behavior on a Likert scale of 1 to 4 (1 = in no way; 4 = very much). The total STAI score ranges between 20 and 80, with a predictive threshold value of anxiety symptoms set at 40. According to this score, it is also possible to define the level of severity: 40 to 50 mild, 50 to 60 moderate, >60 severe [28].

The quality of life was investigated through two “health-related” questionnaires and more precisely:

*EQ5D*: the EuroQol-5 Dimension is a widely used and generic measuring instrument of the quality of life. Short and easy to use in self-administration, this assessment consists of two distinct sections. The first asks for a subjective evaluation of 5 dimensions (mobility, self-care, daily activities, pain/discomfort and anxiety/depression) and each item provides the possibility to choose a level of severity. Each item has graded responses from 1 to 3. Level 1 represents no problem, while level 3 represents extreme limitations. The aggregation of the answers forms a 5-digit number which represents the respondent’s state of health. The 3 response levels, for each of the 5 items, produce a maximum of 243 possible descriptions of the state of health and allow us to highlight the presence/absence of any problems and their intensity [29].

*SF-12* (Short-Form Health Survey) is a short-form generic measure of health-related quality of life. The 12 items are a subset of those in the SF-36, allowing us to describe the state of health through two synthetic indices: PCS (Physical Component Summary) and MCS (Mental Component Summary). PCS concerns the physical state and takes into consideration six questions: two questions concern physical activity, two concern role and physical health, one question is about physical pain, and one is about general health. MCS measures the mental state of the examiner and takes into consideration the other six questions: one question about vitality, one about social activity, two concerning emotional state and two referring to mental health. The normal values are to be considered: 50 ± 10 [30].

Specific questions were added to address changes in lifestyle: “I have been paying attention to my diet”, “I have increased my alcohol consumption”, “I have smoked more”, “I have felt guilty”, “I have been trying to maintain some physical activity every day”, “I have slept badly”, “I have not felt like talking to people”, “I have been scared for my health”. Patients had to refer to the previous 4 weeks, and to respond using a 5-point Likert scale (from 0, indicating never, to 4, indicating always). 

### Statistical Analysis

Statistical analysis was performed with SPSS version 23.0 (SPSS Inc., Chicago, IL, USA). Categorical data are presented as number (percentage) and were compared using the Chi-square test. Continuous data are presented as means (±standard deviation, SD) and were compared with a paired or unpaired Student *t* test as appropriate. *p* Values < 0.05 were considered statistically significant. 

## 3. Results

According to the inclusion and exclusion criteria, the study population consisted of 43 patients, whose characteristics at the three time point visits are reported in Table 1.

At the baseline, mean age was 58 years, 60% of patients were females and all patients presented typical features of metabolic syndrome in 41% of the cases with defined obesity (BMI > 30).

At the time of the first evaluation (T0), 34 patients (79%) were already receiving at least one drug for one of the features of MetS, namely:

- 16 were on hypoglycemic therapy
- 19 received hypolipemic drugs
- 24 received anti-hypertensive drug

A total of 20 patients (46.5% of the cohort, 58.8% of treated subjects) were receiving at least two different drugs for MetS, while 8 (18.6%) were treated with antidepressants.

At the pre-pandemic visit (T1), the entire cohort of our patients showed no significant differences compared to the previous evaluation in anthropometric parameters (BMI, visceral adiposity), in most serum laboratory values, non-invasive fibrosis assessment scores or drug treatment. At variance, there was an increase in physical activity score (1.5 to 1.7, *p* = 0.02), a decrease in serum GGT levels (91.8 to 55.9 U/L, *p* = 0.01) without prescription of choleretic drugs and a trend towards a reduction in liver stiffness (2D-SWE 10.4 to 9.3 kPa, *p* = 0.07).

At the post-lockdown visit (T2), compared to T1, we did not observe a significant worsening of the clinical conditions of our whole cohort, which were stable across clinical, laboratory and elastometric parameters, with the only exception of an increase in visceral adipose tissue thickness (67.6 to 75.4 mm, *p* = 0.007) despite a registered increase in self-reported physical activity (1.7 to 1.9, *p* = 0.04).

Comparing results from T0 and T2, no significant differences were detected aside from an increase in physical activity in the 4 weeks prior to the visit (1.5 to 1.9, *p* = 0.001), an increase in visceral adiposity (66.5 to 75.4 mm, *p* = 0.004) and a decrease in serum levels of gammaGT (91.8 to 59.2 U/L, *p* = 0.01).

At T0, we gave our patients lifestyle and dietary advice, as it is advocated by international guidelines, with the purpose of obtaining at least a 5% weight loss from their initial weight (Supplement Materials Table S1).

Patients were then sub-grouped according to their capacity to reach the weight loss goal before the occurrence of the COVID-19 pandemic, assessed as changes between T0 and T1.

- Group A (>5% of basal weight loss): 9/43 patients (21%) with a mean weight change of −7.5%.
- Group B (<5% of basal weight loss): 34/43 patients (79%) with a mean weight change of +1.2%.

As shown in Table 2, the two groups were homogenous at baseline conditions for most of the variables analyzed, except for body weight (but not BMI) and total serum cholesterol levels, which were significantly higher in Group B, and visceral adiposity, which was slightly higher in group A.

Table 3 shows the characteristics of Groups A and B across the whole study period.

Group A: at T1, patients of Group A showed a significant improvement in all anthropometric parameters compared to T0, with a significant decrease in BMI (29.2 to 27.1, *p* < 0.001), weight (73.9 kg to 68.0 kg, *p* < 0.001), waist circumference (105.6 cm to 97.8 cm, *p* = 0.03) and visceral adiposity (82.8 mm to 68.5 mm, *p* = 0.02), together with an increase in physical activity (1.2 to 1.7, mean increase of 42%, *p* = 0.04). The LDL cholesterol serum levels increased from T0 to T1 but always remained within normal values (81.2 mg/dL to 105.5 mg/dL, *p* = 0.02). The higher number of patients receiving drugs for dyslipidemia reflects the new diagnosis made, and therapies given, at first visit in our clinic, whereas the reduction by one unit in the count of patients needing anti-hypertensive therapy is the case of a man in which pharmacological treatment of hypertension was no longer needed after a significant weight loss (−8.1%). At T2, patients in group A maintained substantial stable anthropometric features compared to T1, confirming a significant reduction compared to T0 in BMI (29.2 to 27.3, *p* = 0.002), weight (73.9 kg to 69.1 kg, *p* < 0.001) and waist circumference (105.6 cm to 96.5 cm, *p* = 0.01), together with a further increase in physical activity (1.8 vs. 1.2, *p* = 0.05). These modifications were associated with an improvement of liver stiffness which did not reach statistical significance but nonetheless marked a clear trend towards reduction (12.8 vs. 13.8 kPa, *p* = 0.055). Between T1 and T2, we did not register any new diagnosis of conditions correlated to MetS.

Group B: at T1, patients of group B showed a decrease in serum GammaGT levels (97.9 U/L to 58.6 U/L, *p* = 0.02) compared to T0. As in group A, also in group B we observed an increase in the number of patients receiving therapy for either diabetes or hypertension due to new diagnosis made during first visit. Compared to T0, at T2 patients in group B showed a worsening of most anthropometric features, with an increase in BMI (29.0 vs. 29.6, *p* = 0.04), weight (78.6 kg to 80.4 kg, *p* = 0.06) and visceral adiposity (63.4 mm to 75.7 mm, *p* < 0.001) despite a self-reported increase in physical activity compared to T0 (*p* = 0.007) and T1 (*p* = 0.005) and a stable reduction in gammaGT levels compared to T0 (97.7 U/L to 58.0 U/L, *p* = 0.01). Two patients in this B group developed dyslipidemia during the observation period.

During the whole observation period of our study, 28 patients changed their pharmacological therapy for diseases correlated to NAFLD and MetS: in 1 case anti-hypertensive therapy was fully stopped, in 1 case statin dosage was persistently reduced, in 9 cases patients received divergent modifications (i.e., a reduction in hypolipemic drug but an increase in hypoglycemic therapy) and the majority of our cohort (17 patients) had to enhance the therapy for NAFLD-associated conditions (either as increase in the dosage of an already ongoing drug or addition of a new medication), in particular:

- 17 patients modified lipid lowering therapy: 2 received a different drug, 13 added a second molecule, and 2 reduced the dosage of a previous therapy
- 9 patients modified antidiabetic therapy: 4 reduced the oral antidiabetic level, 4 increased it one 1 patient was newly prescribed insulin therapy
- 18 patients modified anti-hypertensive therapy: 4 reduced it and 14 incremented it.

Both patients reducing pharmacological therapy belonged to group A and at T1 had shown weight reductions of 8.1% and 9.7%.

Regarding psychoactive therapy, 9 patients experienced some modifications during the observation period: 2 patients could reduce the dose, 6 patients increased it and the addition of a new molecule was needed in 1 case. 

### Psychological Wellbeing and Quality of Life

A total of 14 patients from is presented the total sample fully completed the online survey (33% of the total cohort; this sample comprised 3 of the group A (33.3%) and 11 in group B (32.4%). We report descriptive statistics as the groups are too small for a meaningful statistical comparison.

According to the SRQ-20 test, we identified 3 patients, all belonging to group B, as “positive cases”. In particular, one patient responded affirmatively to all the items, suggesting a moderate psychological suffering which required medical intervention (the patient received a change in antidepressant therapy after lockdown period).

STAI test showed 1 patient with mild anxiety in Group A (33.3%), while in group B 2 patients reported mild and 2 moderate anxiety (36.3%).

The EQ5D questionnaire regarding quality of life recorded data on the following issues:

- Mobility: 4 out of 14 patients, all belonging to group B, affirmed that they felt at least a moderate restriction of their mobility (0% in group A versus 36.3% in group B).
- Self-care: 1 patient in group B reported a decrease in the ability to take care of himself.
- Daily activities: 2 patients in group B stated that they felt a moderate difficulty to routinely perform their daily activities.
- Pain or discomfort: 4 patients declared a moderate pain feeling, with 3 of them being from group B and 1 from group A. One more patient from group B reported severe pain.
- Anxiety or depression: 8 patients reported anxiety or moderate depression, with 6 of them being included in group B and 1 of them suffered from severe depression and being treated with another antidepressant molecule at the post-pandemic visit.

The SF-12 test demonstrated that 2 patients reported low scores in PCS (i.e., physical health including body functioning, ability of performing activity of daily living, and pain), while 3 patients reported low score sin MCS (i.e., mental health including lack of energy, ability to social functioning, depressed mood). All these patients belonged to Group B. 

None of the 3 patients in group A showed low quality of life due to physical limitations or psychological distress. 

Since one patient reported abnormal scores for both fields, a total of 4 patients showed some abnormalities in group B (36.3%) compared to 0 in group A (Table 4).

Separately, we analyzed NAFLD-related specific questions, focusing on the 4 weeks prior to the filling of the questionnaires (Table 5): no significant differences were found in physical health issues (questions 1–4: attention to dietary regime, smoking and drinking habits, physical activity), whereas patients in Group B reported worse results regarding mental status related issues (questions 5–8). In particular, 24 answers from patients in group B (and only one from patients in group A, *p* = 0.004) suggested the presence of greater worries concerning their health status, greater feelings of loneliness, isolation and lower motivation to pursue a healthy diet and a salutary lifestyle.

## 4. Discussion

The SARS-CoV2 pandemic exploded worldwide during the first months of 2020, with terrible consequences that we are still currently observing. 

During the first months of the pandemic, the Italian Government, like many other Governments all around the World, emanated emergency laws and policies which included limitations to personal freedom and social interactions (lockdown), aimed at attempting to limit the spread of SARS-CoV2 infection.

Between March 2020 and May 2020, the lockdown determined a drastic reduction in physical interpersonal relationships (i.e., distance learning for schools of all types and levels, smart-working for everyone except for health-care professionals and other strategic jobs, closure of every recreational, sport-related or commercial activity with the exception of basic necessity stores, abolition of visits to hospitalized patients, etc.). Lockdown measures included a temporary suspension of non-urgent and non-oncological outpatient visits to both concentrate the available resources of Italian National Health Service on the pandemic emergency and to avoid the access of patients to hospitals, where COVID infection had a very high prevalence, unless absolutely necessary. The social distancing measures adopted in the following months, until early 2021, allowed for the progressive re-taking care of outpatients, but continued to affect the daily habits of Italians in many aspects. The drastic changes in lifestyle brought about by these measures, characterized by a reduction in physical activity, a change in eating habits (with an increase in the consumption of snacks and fast-food and a reduction in fresh food, meat and fish) and an increase in perceived stress, have been associated with significant changes in the physical and mental health state of different groups of subjects examined [23,31]. These modifications were found to be associated with a significant but extremely variable weight gain in the general population (from 7 to 72% of cases in a recent systematic review which considered over 460,000 subjects worldwide), alongside a growth of reported conditions of anxiety, depression and insomnia [32,33].

Data available in NAFLD Italian patients showed a weight gain in 48–70% of cases in two different cohorts of subjects (one multicenter study on 357 subjects and one single-center series of 41 patients) evaluated before the advent of the pandemic and immediately after the end of the lockdown, together with a worsening in glucose metabolism, especially in patients who had reported poor adherence to proposed lifestyle and dietary recommendations [24,33].

Our study analyzed in detail the mid-term effects of the lockdown period on the clinical, anthropometric, psychological and lifestyle characteristics of a cohort of 43 patients with non-advanced liver disease in the charge of our NAFLD outpatient clinic. We only included patients in our analysis with complete medical data at three different time points: (1) the first visit (T0) when patients received advices for lifestyle and diet modifications to counteract Metabolic Syndrome and NAFLD, (2) the pre-COVID visit (T1) at least one year after the first one to evaluate the adherence of patients to the physician suggestions and their efficacy, (3) the post-COVID visit (T2) at least 6 months after the end of the lockdown. We decided not to evaluate our patients immediately after the lockdown as in previous studies, but to wait at least after 6 months and thus test with a median total follow-up period of 4 years. This is in light of the long natural history of NAFLD which is scarcely influenced by short-term behavioral changes but rather by prolonged modifications. This prolonged period between pre- and post-COVID visits allowed us to measure the global impact of lockdown on our cohort, including the delaying of the scheduled annual evaluation due to lockdown restrictions.

At the first follow up visit before the COVID-19 advent (T1), our patients demonstrated substantial stability in weight, visceral adiposity, laboratory test and noninvasive fibrosis score, despite a significant increase in physical activity associated with a reduction in gammaGT serum levels without choleretic agents and a trend to reduction in liver stiffness values (from 10.4 kPa to 9.3 kPa, *p* = 0.07).

Overall, our cohort of patients did not demonstrate a significant worsening of their clinical condition following the lockdown period: anthropometric, serum laboratory values and non-invasive test for liver fibrosis remained stable compared to T1, except for a slight thickening of visceral adiposity despite a further increase in physical activity. This phenomenon may be explained by the fact that the assessment of physical activity refers to the last month preceding the visit and not to the whole period from the pre-COVID visit onward. It is thus plausible that our patients had gained visceral adiposity during the lockdown period and increased their physical activity as soon as the social distancing was loosened.

When we analyzed subgroups of patients according to their weight loss at T1, we found a diverging trend in the two populations despite similar characteristics at baseline: those who had reached the weight-reduction goal of >5% since first visit (group A), assumed to be the ones able to effectively adopt the lifestyle changes proposed, maintained this result even through the lockdown period, eventually achieving a reduction in liver stiffness measured by 2D-SWE elastometry and, in 2 cases (22%), a stable reduction in metabolic-oriented pharmacological therapy; instead, those who had not achieved a significant weight loss (group B), assumed to correspond to persons unable to incorporate effective lifestyle changes, showed a further weight gain through the COVID pandemic (on average 10% compared to pre-COVID evaluation), though it was lower than that reported by other authors [23,31].

To shed some light on whether the inability to adopt effective lifestyle changes was connected with frailty in facing stressful conditions such as the COVID-19 lockdown, we examined the psychological wellbeing and quality of life in a subgroup of patients. Our data do not allow us to define with sufficient statistical power whether and to what extent the lockdown has impacted the psychological health and quality of life, and thus the physical health of our patients. However, some trends of clinical interest that require future investigations have been identified. In particular, using both the validated tests and our lifestyle-specific questions, we observed a trend for more prominent alterations in psycho-physical health status, especially in the affective domain of anxiety, depression, and somatization in the group of patients who were not able to adopt lifestyle changes leading to weight loss. This aspect may have been partly affected by the worsening of the underlying metabolic conditions in group B, though it was not limited to subjects who were newly diagnosed with diabetes or hypertension.

The major limitations of our study reside in the small percentage of patients who agreed to participate and subsequently completed the online questionnaire thoroughly. This poor collaboration, due to the concurrence of several factors including lack of familiarity with internet technology in a population of middle aged–elderly patients, would certainly have been increased if the questionnaire had been administer face to face during the outpatient visit. Unfortunately, the lockdown condition itself, whose effects we wanted to analyze, forced us to use a remote approach with all the consequent limitations. Further larger studies will be necessary to evaluate the effects of psychological frailty on the ability to adopt effective lifestyle changes in patients with NAFLD and metabolic syndrome.

## 5. Conclusions

Our study aimed to analyze the effects of the SARS-CoV2-related lockdown on a cohort of outpatients with non-evolved NAFLD. The establishment of an effective counselling at the first clinical evaluation, followed by a strengthening at the pre-pandemic visit, was effective in contrasting lockdown effects in terms of weight gain and the worsening of the metabolic profile. Nonetheless, as NALFD and metabolic syndrome are multifaceted conditions, a multidisciplinary approach based on the cooperation of clinicians, psychologists, nutritionists and specialized trainers, even with the help of new technologies such as telemedicine or specifically developed apps, is needed to counteract them. This is especially the case in the presence of coexisting mild psychological disorders that can severely impair the adherence of the patient to lifestyle modifications and dietary regime, which still represent the cornerstones of NAFLD treatment.

## Figures and Tables

**Table 1:** Characteristics of the entire population (*n* = 43) at three different time point visits.

Parameters	First Visit (T0)	Pre-Pandemic Visit (T1)	Post-Pandemic Visit (T2)	*p*
Sex M (%)	17 (40%)	17 (40%)	17 (40%)	n.s.
Diabetes *n* (%)	15 (34.9%)	18 (41.9%)	18 (41.9%)	n.s.
Hypertension *n* (%)	27 (62.8%)	27 (62.8%)	27 (62.8%)	n.s.
Dyslipidemia *n* (%)	25 (58.1%)	28 (65.1%)	30 (69.8%)	n.s.
Weight (kg)	77.6 ± 10.9	77.1 ± 11.8	78.0 ± 12.3	n.s.
BMI	29.0 ± 4.2	28.8 ± 4.3	29.1 ± 4.7	n.s.
Waist circumference (cm)	105.3 ± 13.3	103.1 ± 12.0	101.3 ± 10.4	*c*
Physical activity	1.5 ± 0.6	1.7 ± 0.6	1.9 ± 0.8	*a,b,c*
Triglycerides (mg/dL)	133.4 ± 62.7	123.4 ± 50.6	145.8 ± 71.4	n.s.
Total cholesterol (mg/dL)	194.1 ± 40.2	188.6 ± 34.3	188.1 ± 43.9	n.s.
HDL (mg/dL)	54.6 ± 23.8	51.9 ± 12.9	57.3 ± 31.6	n.s.
LDL (mg/dL)	111.9 ± 37.8	111.4 ± 29.9	108.6 ± 45.3	n.s.
HOMA index	3.2 ± 2.5	3.3 ± 2.1	4.1 ± 3.9	n.s.
ALT/GPT (U/L)	47.6 ± 27.2	38.8 ± 27.4	40.3 ± 27.7	n.s.
AST/GOT (U/L)	37.7 ± 15.2	33.0 ± 16.1	34.5 ± 17.5	n.s.
gammaGT (U/L)	91.8 ± 80.8	55.9 ± 40.4	59.2 ± 44.3	*a,c*
NAFLD fibrosis score	−0.80 ± 1.68	−0.58 ± 2.07	−0.45 ± 1.97	n.s.
FIB-4	1.73 ± 1.00	1.77 ± 1.84	1.91 ± 1.52	n.s.
Visceral adiposity (mm)	66.5 ± 23.5	67.6 ± 21.7	75.4 ± 22.2	*b,c*
Hepato-renal Sonographic Index	1.8 ± 0.6	1.9 ± 0.8	1.8 ± 0.6	n.s.
2D-ShearWave elastography (kPa)	10.4 ± 8.4	9.3 ± 7.3	10.3 ± 10.4	n.s.
Patients receiving anti-hypertensive drugs *n* (%)	24 (55.8%)	28 (65.1%)	28 (65.1%)	n.s.
Patients receiving hypoglycemic drugs *n* (%)	16 (37.1%)	17 (39.5%)	17 (39.5%)	n.s.
Patients receiving hypolipemic drugs *n* (%)	19 (44.2%)	25 (58.1%)	27 (62.8%)	n.s.
Patients receiving antidepressants *n* (%)	8 (18.6%)	7 (16.3%)	11 (25.6%)	n.s.

Data are expressed as absolute numbers (%) or mean ± standard deviation. *a: p* <0.05 between T0 and T1; *b*: *p* < 0.05 between T1 and T2; *c*: *p* < 0.05 between T0 and T2.

**Table 2:** Group A and Group B characteristics at first visit (T0).

Variables	Group A T0 (*n* = 9)	Group B T0 (*n* = 34)	*p*
Age	62.0 ± 12.0	56.9 ± 11.9	n.s.
Sex M (%)	3 (33.3%)	14 (41.1%)	n.s.
Diabetes *n* (%)	4 (44.4%)	11 (32.4%)	n.s.
Hypertension *n* (%)	6 (66.7%)	21 (61.8%)	n.s.
Dyslipidemia *n* (%)	6 (66.7%)	19 (55.9%)	n.s.
Weight (kg)	73.9 ± 13.4	78.6 ± 9.9	0.008
BMI	29.2 ± 3.0	29.0 ± 4.5	n.s.
Waist circumference (cm)	105.6 ± 7.0	105.2 ± 17.9	n.s.
Physical activity	1.2 ± 0.4	1.5 ± 0.7	n.s.
Triglycerides (mg/dL)	157.8 ± 77.0	128.7 ± 59.8	n.s.
Total cholesterol (mg/dL)	161.7 ± 26.9	200.9 ± 39.4	0.01
Cholesterol HDL (mg/dL)	45.3 ± 14.4	56.5 ± 25.1	n.s.
Cholesterol LDL (mg/dL)	81.2 ± 40.2	118.0 ± 34.7	0.02
HOMA index	3.8 ± 3.5	3.0 ± 2.5	n.s.
ALT/GPT (U/L)	45.4 ± 21.9	48.1 ± 28.8	n.s.
AST/GOT (U/L)	35.9 ± 12.4	38.2 ± 16.1	n.s.
gammaGT (U/L)	63.2 ± 54.7	97.9 ± 85.0	n.s.
NAFLD fibrosis score	−1.1 ± 1.7	−0.7 ± 1.7	n.s.
FIB-4	1.99 ± 1.46	1.65 ± 0.83	n.s.
Visceral adiposity (mm)	82.8 ± 24.6	63.4 ± 22.4	0.09
Hepato-renal Sonographic Index	1.8 ± 0.5	1.8 ± 0.7	n.s.
2D-ShearWave elastography (kPa)	13.8 ± 10.5	9.4 ± 7.5	n.s.
Patients receiving anti-hypertensive drugs *n* (%)	5 (55.6%)	19 (55.9%)	n.s.
Patients receiving hypoglycemic drugs *n* (%)	4 (44.4%)	12 (35.3%)	n.s.
Patients receiving hypolipemic drugs *n* (%)	4 (44.4%)	15 (44.1%)	n.s.
Patients receiving antidepressant *n* (%)	2 (22.2%)	6 (17.7%)	n.s.

Data are expressed absolute numbers (%) or mean ± standard deviation.

**Table 3:** Group A and Group B characteristics across the whole length of the study (T0-T1-T2).

Variables	Group A			*p*	Variables	Group B			*p*
	T0	T1	T2			T0	T1	T2	
Sex M (%)	3 (33.3%)	3 (33.3%)	3 (33.3%)		Sex M (%)	14 (41.1%)	14 (41.1%)	14 (41.1%)	
Diabetes *n* (%)	4 (44.4%)	4 (44.4%)	4 (44.4%)		Diabetes *n* (%)	11 (32.4%)	14 (41.2%)	14 (41.2%)	
Hypertension *n* (%)	6 (66.7%)	5 (55.5%)	5 (55.5%)		Hypertension *n* (%)	21 (61.8%)	22 (64.7%)	22 (64.7%)	
Dyslipidemia *n* (%)	6 (66.7%)	8 (88.9%)	8 (88.9%)		Dyslipidemia *n* (%)	19 (55.9%)	20 (58.8%)	22 (64.7%)	
Weight (kg)	73.9 ± 13.4	68.0 ± 12.4	69.1 ± 12.8	*a,c*	Weight (kg)	78.6 ± 9.9	79.4 ± 9.4	80.4 ± 9.3	
BMI	29.2 ± 3.0	27.1 ± 2.8	27.3 ± 3.0	*a,c*	BMI	29.0 ± 4.5	29.3 ± 4.9	29.6 ± 4.9	*c*
Waist circumference (cm)	105.6 ± 7.0	97.8 ± 11.1	96.5 ± 9.0	*a,c*	Waist circumference (cm)	105.2 ± 17.9	104.6 ± 10.4	103.0 ± 10.8	
Physical activity	1.2 ± 0.4	1.7 ± 0.5	1.8 ± 0.8	*a,c*	Physical activity	1.5 ± 0.7	1.7 ± 0.6	1.9 ± 0.8	*b,c*
Triglycerides (mg/dL)	157.8 ± 77.0	122.0 ± 43.6	148.1 ± 56.2		Triglycerides (mg/dL)	128.7 ± 59.8	123.8 ± 52.9	145.1 ± 76.3	
Total cholesterol (mg/dL)	161.7 ± 26.9	178.2 ± 51.3	175.6 ± 40.0		Total cholesterol (mg/dL)	200.9 ± 39.4	191.5 ± 28.2	191.7 ± 44.9	
Cholesterol HDL (mg/dL)	45.3 ± 14.4	50.0 ± 15.9	50.6 ± 15.0		Cholesterol HDL (mg/dL)	56.5 ± 25.1	52.4 ± 12.1	59.0 ± 34.7	
Cholesterol LDL (mg/dL)	81.2 ± 40.2	105.5 ± 28.8	106.6 ± 30.6	*a*	Cholesterol LDL (mg/dL)	118.0 ± 34.7	113.0 ± 30.6	109.0 ± 48.9	
HOMA index	3.8 ± 3.5	3.0 ± 1.3	3.1 ± 1.7		HOMA index	3.0 ± 2.5	3.5 ± 2.3	4.3 ± 4.2	
ALT/GPT (U/L)	45.4 ± 21.9	40.8 ± 24.8	43.3 ± 33.3		ALT/GPT (U/L)	48.1 ± 28.8	38.2 ± 28.4	39.5 ± 26.6	
AST/GOT (U/L)	35.9 ± 12.4	35.0 ± 12.5	35.0 ± 13.6		AST/GOT (U/L)	38.2 ± 16.1	32.5 ± 17.1	34.3 ± 18.6	
gammaGT (U/L)	63.2 ± 54.7	43.6 ± 31.7	64.4 ± 66.2		gammaGT (U/L)	97.9 ± 85.0	58.6 ± 42.2	58.0 ± 40.0	*a,c*
NAFLD fibrosis score	−1.1 ± 1.7	−1.3 ± 1.7	−1.1 ± 1.5		NAFLD fibrosis score	−0.7 ± 1.7	−0.4 ± 2.2	−0.3 ± 2.0	
FIB-4	1.99 ± 1.46	1.50 ± 0.71	1.62 ± 0.79		FIB-4	1.65 ± 0.83	1.8 ± 2.0	1.97 ± 1.64	
Visceral adiposity (mm)	82.8 ± 24.6	68.5 ± 19.8	73.8 ± 20.3	*a*	Visceral adiposity (mm)	63.4 ± 22.4	67.4 ± 22.3	75.7 ± 22.8	*b*
Hepato-renal Sonographic Index	1.8 ± 0.5	2.0 ± 1.3	1.7 ± 0.4		Hepato-renal Sonographic Index	1.8 ± 0.7	1.8 ± 0.7	1.8 ± 0.6	
2D-ShearWave Elastography (kPa)	13.8 ± 10.5	12.9 ± 11.4	12.8 ± 11.3		2D-ShearWave Elastography (kPa)	9.4 ± 7.5	8.3 ± 5.5	9.6 ± 7.0	
Patients receiving anti-hypertensive drug *n* (%)	5 (55.6%)	4 (44.4%)	4 (44.4%)		Patients receiving anti-hypertensive drug *n* (%)	19 (55.9%)	22 (64.7%)	23 (67.6%)	

Data are expressed absolute numbers (%) or mean ± standard deviation. *a: p* < 0.05 between T0 and T1; *b*: *p* < 0.05 between T1 and T2; *c*: *p* < 0.05 between T0 and T2.

**Table 4:** Heat map of psychological health and quality of life.

	SRQ-20	EQ-5D					SF-12		STAI
		I	II	III	IV	V	PCS-12	MCS-12	
Group A	6	1	1	1	1	2	53.0	49.9	43
	6	1	1	1	2	2	47.1	55.9	
	1	1	1	1	1	1	55.5	57.8	
Group B	3	1	1	1	2	1	48.0	59.6	
	1	1	1	1	1	2	54.2	56.0	
	9	1	1	1	1	2	55.8	37.1	50
	4	1	1	1	1	1	49.6	57.4	
	8	1	1	1	1	3	55.8	28.1	42
	5	1	1	1	1	2	48.1	53.8	42
	3	2	1	2	2	1	29.3	56.9	
	6	2	2	1	2	2	40.4	56.4	
	12	2	1	2	3	2	29.1	25.7	56
	0	1	1	1	1	1	55.9	55.9	
	5	1	1	1	1	2	47.5	51.7	

Each row represents one patient. Color intensity represents the severity of the disease according to validated cut-off, namely: SRQ-20 >7 indicates psychological suffering; EQ-5D: 1 = no symptoms; 2 = moderate; 3 = severe life quality affection; SF-12: normal values for physical and mental health >40; STAI: anxiety if >40, with the following classes: between 40 and 50 = mild anxiety; between 50 and 60 = moderate anxiety; >60 = severe anxiety.

**Table 5:** NAFLD related specific questions, personally elaborated for this study.

	Group A					Group B				
In the Last 4 Weeks	Never	Rarely	Sometimes	Often	Always	Never	Rarely	Sometimes	Often	Always
I have paid attention to my diet		1	2			1	3	4	3	
I have increased my alcohol consumption	2	1				9	2			
I have smoked more	3					10	1			
I have felt guilty	1	1	1			7	3	1		
I have tried to maintain some physical activity every day	1	1		1			3	2	1	5
I have slept badly	1	1	1			2	1	6	1	1
I have not felt like talking to people	3					4		4	3	
I have been scared for my health		3				3	3	2	2	1
I have felt less motivated to follow healthy behaviors	2	1				4	3	1	3	

Data are expressed as absolute numbers of patients who selected the answers. Color intensity represents the severity of the disease.

## Data Availability

The data presented in this study are available on request from the corresponding author. The data are not publicly available due to privacy reasons.

## Supplementary Materials

The following supporting information can be downloaded at: https://www.mdpi.com/article/10.3390/nu15061445/s1, Table S1: Lifestyle indications for patients with NAFLD.
