# Supplementary material for: Effects of COVID-19 Pandemic on Metabolic Status and Psychological Correlates of a Cohort of Italian NAFLD Outpatients
Source: Nutrients. 2023 Mar 16;15(6):1445. doi: 10.3390/nu15061445 (PMC10052521; doi:10.3390/nu15061445)
Supplement: Supplementary file 1 [file nutrients-15-01445-s001.zip › nutrients-2259446-supplementary.pdf]

Table S1. LIFESTYLE INDICATIONS FOR PATIENTS WITH NAFLD.

1. Eliminate sugary drinks including sodas, fruit juices, iced tea and other beverages with added sugars.
2. Reduce as much as possible the consumption of ultra-processed foods (pre-cooked foods, confectionery and baked goods, ice cream, canned fruit).
3. Reduce the consumption of highly refined cereals and flours favoring wholemeal flours and whole gain cereals, if possible of organic origin.
4. Consume vegetables both at lunch and dinner (limit potatoes), preferably fresh and in season, both raw or cooked; consume fruit both at lunch and dinner (limit bananas), if tolerated at the end of the meal.
5. Consume at least 4 portions of fish a week, even frozen or canned (for example tuna or mackerel in oil, well drained).
6. Prefer cooking methods at low temperatures and limit cooking with direct contact between heat source and the food as for example on the grill.
7. Eliminate vegetable margarines. As raw condiment, use extra virgin olive oil or cold—pressed linseed oil, all spices are allowed. When cooking, use extra virgin olive oil or little amounts of butter, better if clarified.
8. Introduce a portion (20-25 g) of nuts (walnuts, almonds or hazelnuts) or seeds every day, during the main meals or as a snack but not after dinner.
9. As dessert, a small amount of dark chocolate (>80%) is allowed.
10. The consumption of maximum one unit (125 ml) of wine per day is allowed, eliminate other types of alcoholic drinks.
11. In case of diabetes or insulin resistance, start the meal with fibers and proteins, consuming carbohydrates last.
12. Practice physical activity at moderate intensity, both aerobic and anaerobic, for at least 150 minutes per week, favoring outdoor activities with sunlight exposure.
13. Promote a regular night rest (at least 7 hours).
14. Try to ensure an overnight fasting interval of at least 12 hours between the end of dinner and the beginning of breakfast.
